# Single cell imaging and quantification of TDP-43 and α-synuclein intercellular propagation

**DOI:** 10.1038/s41598-017-00657-z

**Published:** 2017-03-28

**Authors:** Sivan Peled, Dorin Sade, Yaron Bram, Ziv Porat, Topaz Kreiser, Michael Mimouni, Alexandra Lichtenstein, Daniel Segal, Ehud Gazit

**Affiliations:** 1 Department of Molecular Microbiology and Biotechnology, Tel-Aviv University, Tel Aviv, 6997801 Israel; 2 Flow Cytometry Unit, Department of Biological Services, Weizmann Institute of Science, Rehovot, Israel; 3 Department of Ophthalmology, Rambam Health Care Campus, Haifa, Israel; 4 Sackler Cellular and Molecular Imaging Center, Sackler Faculty of Medicine, Tel-Aviv University, Tel Aviv, Israel; 5 Sagol Interdisciplinary School of Neurosciences, Tel Aviv University, Tel Aviv, 6997801 Israel; 6 Division of Gastroenterology & Hepatology, Department of Medicine, Weill Medical College of Cornell University, New York, NY 10021 USA

## Abstract

The intercellular spreading of protein assemblies is a major factor in the progression of neurodegenerative disorders. The quantitative study and visualization of cell-to-cell propagation using tagged-proteins is challenging due to the steric effect of relatively large fluorescence tags and the risk of ‘false positive’ identification when analyzing these rare transmission events. Here, we established a cell culture model to characterize the cell-to-cell transmission of TAR DNA-binding protein and α-synuclein, involved in amyotrophic lateral sclerosis and Parkinson’s disease, respectively, using the small nine amino acid influenza hemagglutinin tag. The novel use of single cell resolution imaging flow cytometry allowed the visualization and quantification of all individual transmission events. Cell-level analysis of these events indicated that the degree of transfer is lower than previously reported based on conventional flow cytometry. Furthermore, our analysis can exclude ‘false positive’ events of cellular overlap and extracellular debris attachment. The results were corroborated by high-resolution confocal microscopy mapping of protein localization.

## Introduction

Neurodegenerative diseases include a large group of pathological conditions in which progressive degeneration and dysfunction of neurons occur in affected regions of the central nervous system (CNS). Although these conditions are clinically diverse, the majority of the disorders share a key common neuropathological feature of intracellular or extracellular disease-related protein accumulation and deposits^1, 2^. Disease progression is assumed to be initiated by protein misfolding followed by amyloidal self-assembly of an extensive variety of pathological proteins and polypeptides^3^, such as β-amyloid and tau in Alzheimer’s disease (AD)^4, 5^, α-synuclein in Parkinson’s disease (PD)^6^, TAR DNA-binding protein (TDP-43) in amyotrophic lateral sclerosis (ALS)^7^ and the prion protein in Creutzfeldt-Jakob disease^8^. Accumulating evidence suggests that these pathologies spread in a stereotypical pattern in the human brain, a process that most likely relies on cell-to-cell transmission of the pathological proteins^9–12^. Since the mechanisms underlying the formation and propagation of aggregates in the CNS remain unclear, investigation of the phenomenon of amyloidogenic proteins spreading is at the forefront of current research. The similarities between the propagation of amyloidogenic protein assemblies and infectious prion proteins, as in the case of bovine spongiform encephalopathy, suggest that a common spreading mechanism may exist. The implications of this stereotypical process are fundamental both for understanding the etiology of these diseases as well as for the development of therapeutic intervention.

PD is the second most common form of neurodegenerative diseases, after AD, affecting 1–2% of the elderly population with no disease-modifying therapy currently available^13^. Recent studies described prion-like spreading of misfolded α-synuclein^14^. This process has been proposed to contribute to the propagation of the PD-characteristic Lewy body inclusions throughout the nervous system in affected individuals. The dynamic distribution pattern of α-synuclein aggregates in the CNS is well documented^15^. The aggregative forms first appear in stem nuclei of the lower brain, and spread sequentially into the midbrain, followed by mesocortical and neocortical regions^16^. Neural grafting experiments^17, 18^ and cell culture models^19, 20^ support the notion that α-synuclein undergoes intercellular transfer and seeds pathological aggregates in a prion-like fashion. Furthermore, accumulating evidence supports the transfer of α-synuclein from the gastro-intestinal track to the brain via the peripheral nervous system^21^. Therefore, in the case of PD, therapeutic targeting of cell-to-cell transfer of the amyloidogenic protein may be effective even prior to any brain-borne symptoms.

While the intercellular transfer of α-synuclein, tau and β-amyloid has been confirmed^22^, prion-like cell-to-cell transmission of TDP-43, implicated in ALS and fronto-temporal lobar dementia (FTLD), is still to be further substantiated^23^. TDP-43 (wild type) is the major component in cytoplasmatic inclusions in neurons of sporadic ALS^7, 24, 25^. This indicates that a mutation is not necessarily required to trigger the pathological aggregation. The inclusions were reported to be Thioflavin-S (ThS) positive^26^, a feature typical of amyloid assemblies, although TDP-43 amyloidogenicity is still debatable^27–29^. Nevertheless, prion-like properties of TDP-43 were identified in extracts from patient brains^30^. It was recently reported that exposure of neuronal cells to cerebrospinal fluid samples taken from ALS and FTLD patients leads to TDP-43 aggregation mediated by exosomes and tunneling nanotube-like structures^31^. A recent finding in post-mortem brains of ALS patients demonstrated a spreading pattern of phosphorylated TDP-43 between distant areas in the CNS by axonal transport and transmission across synapses^32^. Furthermore, TDP-43 was shown to transmit across axon terminals in a cell-based protein complementation assay^33^.

Given the importance of α-synuclein and TDP-43 in the pathology of neurodegenerative diseases, there is an unmet need to monitor the process of cell-to-cell transmission of these pathological protein assemblies. The extent, efficiency and dynamics of the spreading of α-synuclein, TDP-43, or other amyloidogenic proteins are still not unequivocally determined due to the low frequency of the process, the occurrence of ‘false positive’ events and the heterogeneity between cells. Pioneering work by Brundin and co-workers^19^ described a cell culture model to monitor cell-to-cell transfer, in which cell lines expressing α-synuclein fused to GFP/mCherry or the fluorescent tags alone were co-cultured. They found that 3.5–5.4% of co-cultured GFP cells were double-labeled with mCherry, and suggested that this was a result of mCherry-tagged α-synuclein transfer. However, co-culturing of cells expressing fluorescent tags without the amyloidogenic protein, demonstrated a similar frequency of transfer between cells^19^. Thus, there is a need to confirm the role of the specific amyloidogenic proteins in the observed prion-like behavior, as well as to provide detailed monitoring of the phenomenon at a single cell resolution.

Here we used the short influenza hemagglutinin (HA) tag^34^ to label the amyloidogenic proteins and utilized Imaging Flow Cytometry (IFC) for high throughput acquisition and analysis of protein transmission levels. In IFC, single cells in suspension are illuminated by a set of lasers and a bright field light source and are imaged in flow at high speed by a dedicated CCD camera. Moreover, this allows rapid and statistically robust data acquisition followed by detailed automated and unbiased morphological analysis of the cells^35^. We used IFC to quantify the transfer of α-synuclein and TDP-43 and observed differences in transmission occurrence over time and between the two proteins. We further improved the analysis accuracy by excluding artifacts such as events of debris attachment to the outer cell membrane, demonstrating that the actual transfer level is lower than previously reported^19^. This novel single cell approach may help to elucidate the mechanism of transfer of amyloidogenic proteins and aid in the development of therapeutic agents that could block the pathological spreading.

## Results

**Figure 1:**
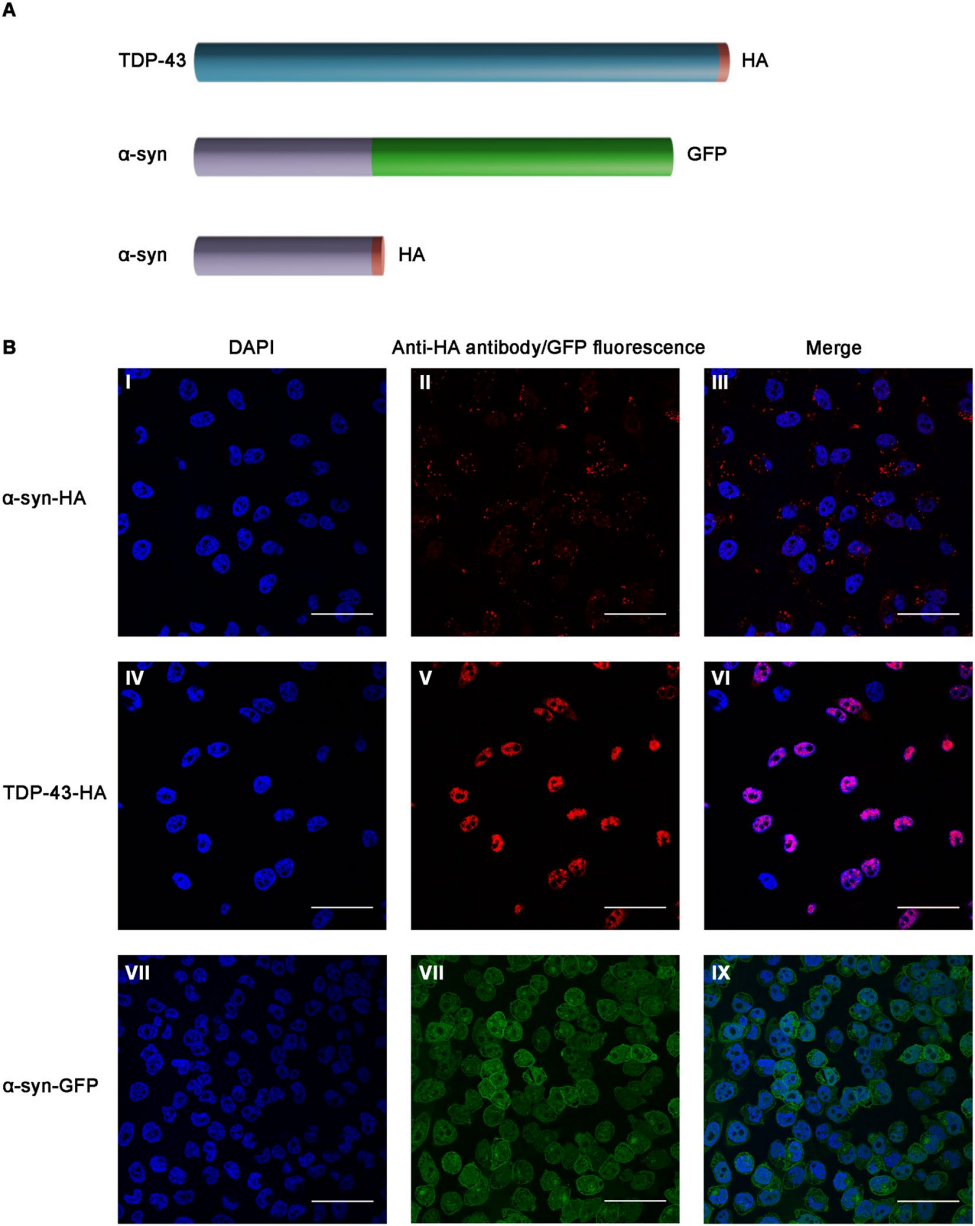
(**A**) Scheme of the studied proteins (α-synuclein and TDP-43) with their fused tags (HA and GFP). (**B**) SH-SY5Y cells stably expressing tagged TDP-43 and α-synuclein. I–III Over-expressed HA-tagged α-synuclein is located in the cytoplasm. IV–VI Over-expressed HA-tagged TDP-43 is located in the nucleus. VII–IX Over-expressed GFP-tagged α-synuclein is located in the cytoplasm. Cells were fixed and stained with DAPI or immuno-stained with a specific HA-antibody and viewed using confocal microscopy. Scale bar: 50 µm.

To provide a practical extension to the use of fluorescent tags, which are actually larger than the studied amyloidogenic proteins, we developed an alternative protein labeling system. We chose the significantly smaller HA tag^34^ which is extensively used and validated. HA is only nine amino acids long (YPYDVPDYA), which likely reduces its effect on the chemical and physical properties of the investigated proteins. This allows to overcome a possible limitation in the use of the traditional GFP tag which is relatively large (238 amino acids) (illustrated in Fig. 1A) and is potentially sensitive to environmental conditions. Studies have demonstrated that labeling α-synuclein with a fluorescent protein, such as EGFP, interferes with protein aggregation, *in vitro* ^36^. The experimental data shows that even though the EGFP does not inhibit the aggregation of the α-synuclein-EGFP fusion, the fibrillation pathway is affected by the conformation of the tag^36^. Additional work demonstrated a drop in the fluorescence for fibrils formed from YFP labelled α-synuclein^37^. However, no comparable experimental work has been reported for TDP-43. Also, as was recently discussed by Viswanathan *et al*.^38^, the small size of epitope tags, such as HA (illustrated in Fig. 1A), enables their attachment to proteins of interest without affecting their folding, targeting or protein-protein interactions.

To monitor TDP-43 and α-synuclein localization and intercellular transmission in neural cells, SH-SY5Y cells stably over-expressing C-terminally HA-tagged TDP-43 and HA/GFP-tagged α-synuclein were used (illustrated in Fig. 1A). We cloned the constructs under a cytomegalovirus (CMV) promoter, leading to constitutive over-expression. Next, we used a lentiviral vector system for constitutive gene expression and selected the infected cells using puromycin. The infected cells did not originate from a specific clone but rather represented an average-heterogeneous population. This strategy is important for the reproducibility and better represents the pathological state as compared to the exclusive selection of only highly expressing infected cells. Figure 1B depicts TDP-43 and α-synuclein expressing cells that were generated. Immunological staining of cells expressing the tagged proteins using an anti-HA antibody allowed specific detection of over-expressed TDP-43 or α-synuclein. The tagged α-synuclein was mainly localized throughout the cell and formed characteristic aggregates apparent as cellular puncta, a pathological hallmark of PD^39^ (Fig. 1B_I–III_). Importantly, the HA-tagged TDP-43 was predominantly nuclear (Fig. 1B_IV–VI_), as reported for endogenous TDP-43^7^, suggesting that the fused protein fully recapitulates the behavior of endogenous TDP-43. Over-expressed GFP-tagged α-synuclein, detected as GFP native fluorescence, was distributed throughout the cell (Fig. 1B_VII–IX_). We quantified the total over-expressed HA-tagged proteins by preparing cell lysates and analyzing the samples by western blot (Supplementary Fig. S1). We found that both proteins are expressed ~3 fold higher compared to the corresponding endogenous protein.

**Figure 2:**
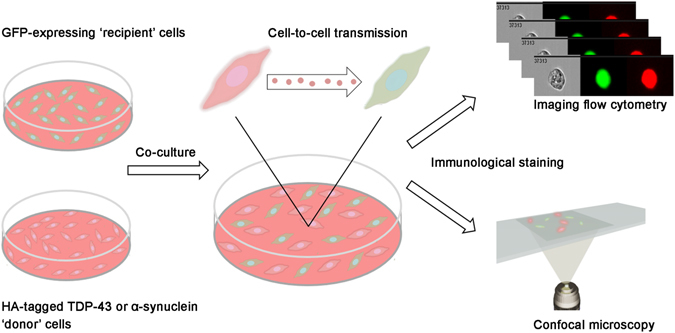
Schematic presentation of the experimental system. ‘Recipient’ cells are co-cultured with ‘donor’ cells for one or three days. During this time, protein transmission can take place. Next, the cells are stained and analyzed using both IFC and confocal microscopy.

Using this cell culture system, we next aimed to visualize and quantify the cell-to-cell transfer of tagged TDP-43 and α-synuclein. We co-cultured ‘recipient’ cells, over-expressing solely GFP, with ‘donor’ cells, over-expressing HA-tagged TDP-43 or α-synuclein, and analysis was conducted using IFC and confocal microscopy (illustrated in Fig. 2).

A major issue in the analysis of protein transmission is the monitoring of a bulk process which represents a very large collection of various single events. In the current work, a single cell level methodology, IFC, was applied allowing the visualization and analysis of individual cells. The cells were co-cultured for either one day or three days and then fixed and immuno-stained using a primary anti-HA antibody and a secondary Cy5 fluorescent antibody. The background baseline was determined by mixing fixed ‘donor’ and ‘recipient’ cells, representing ‘time point zero’. For each time point of co-culture we collected 100,000 focused images of single GFP-expressing ‘recipient’ cells. The pattern and intensity of GFP-expressing ‘recipient’ cells cultured alone served as a reference for validating that only GFP-expressing cells are collected from the co-culture. We defined threshold parameters to avoid the false classification of cell doublets or cell debris attachment that could have been misinterpreted.

**Figure 3:**
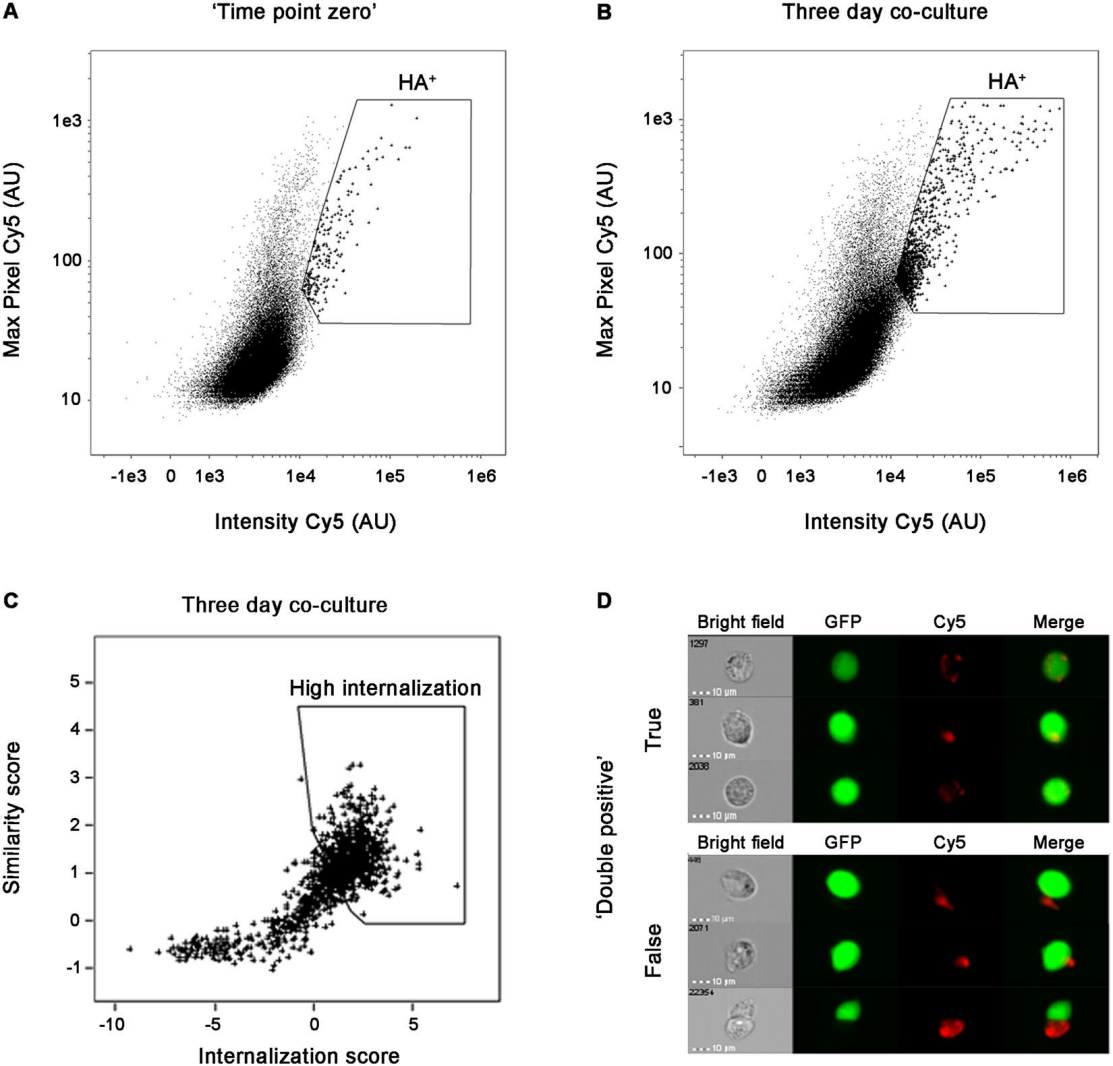
IFC analysis of GFP-expressing ‘recipient’ cells co-cultured with ‘donor’ cells expressing HA-tagged α-synuclein. (**A**,**B**) Bivariate analysis of GFP-expressing ‘recipient’ cells collected from (**A**) ‘time point zero’ and (**B**) three day co-culture. HA^+^ gated cells are demarcated. (**C**) Double positive (HA^+^) cells collected after three days co-culture were further gated to exclude Cy5 staining outside cell boundaries using the Internalization score and the Similarity score between the GFP and Cy5 channels (see Materials and Methods for details). The gated cells represent a high Internalization score within the GFP-expressing cells. (**D**) Images representing true and false ‘double positive’ cells.

Single cell imaging was essential to verify that classification of cells as ‘double positive’ for HA staining and GFP fluorescence indeed represents HA-tagged TDP-43 or α-synuclein transfer to a naïve ‘recipient’ GFP cell. The GFP-expressing cells were gated for positive Cy5 staining, representing α-synuclein (Fig. 3) or TDP-43 (Supplementary Fig. S2) transfer, using the Intensity (total amount of staining fluorescence) and Max Pixel (value of the highest intensity pixels within the image) features. The baseline was set according to ‘time point zero’ (Fig. 3A, Supplementary Fig. S2). This fixed gate (termed: HA^+^) defined the differentially stained cell population in the subsequent time points during the co-culture (Fig. 3B, Supplementary Fig. S2). To eliminate images in which the Cy5 staining was outside the cell boundaries, cells were further gated using two additional features: the Similarity score between the Cy5 and GFP label (Log transformed Pearson’s correlation coefficient between the two images), and the Internalization score (Cy5 intensity within the GFP mask) (Fig. 3C, Supplementary Fig. S2). This fixed gate (termed: high internalization) defines only the cells in which the Cy5 staining is restricted to the intracellular region (see Materials and Methods). Representative single images of these genuine ‘double positive’ cells are presented in Fig. 3D and Supplementary Fig. S2. False ‘double positive’ cases, which are located outside the gate boundaries, were excluded from the quantification; individual images of such events are displayed in Fig. 3D and Supplementary Fig. S2.

**Figure 4:**
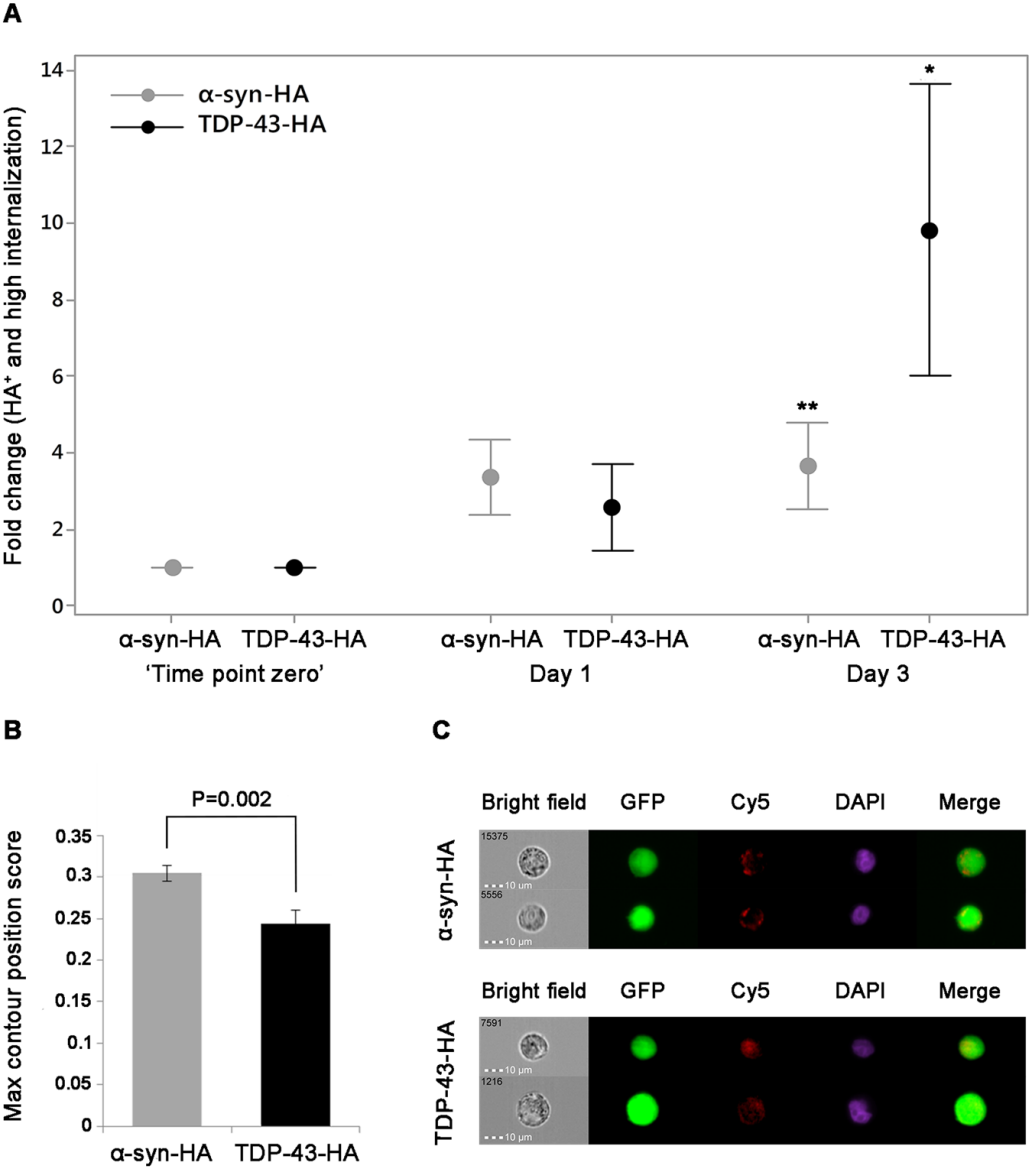
Quantification of transmission events and localization analysis of the HA-tagged α-synuclein and TDP-43 in GFP-expressing ‘recipient’ cells. (**A**) Quantification of transmission events over time presented as fold change ratio compared to the corresponding ‘time point zero’ (P = *0.03**0.005 each compared to ‘time point zero’). (**B**) Mean value of the intracellular localization score calculated using the Max Contour Position score (see Materials and Methods for details). (**C**) Representative images of HA-tagged α-synuclein and TDP-43. DAPI staining pinpoints to the nucleus and facilitates identifying the localization of the Cy5 staining. In each series of experiments n = 8 biological repeats, each comprising 100,000 cells.

IFC IDEAS software was used to calculate the percentage of cells that are ‘double positive’ (HA^+^) according to the criteria we defined (Fig. 3 and Supplementary Fig. S2). To extrapolate the exact number of ‘true’ transmission events, we calculated the percentage of the high internalization gated and HA^+^ gated cells. A significant increase of 0.11–1.74% in ‘recipient’ GFP cells that were genuine ‘double positive’, as compared to ‘time point zero’, was observed after a three day co-culturing with HA-tagged α-synuclein, representing the transmission events. The results are presented in Fig. 4A as fold change ratio compared to ‘time point zero’ for each time point. For TDP-43, there was a significant increase of 0.13–1% in genuine ‘double positive’ GFP cells over a similar period of time (Fig. 4A). On day three both α-synuclein and TDP-43 demonstrated a significant rise (P = 0.005 and P = 0.03, respectively) in transmission events compared to ‘time point zero’ (repeated-measures analysis of variance [ANOVA] followed by Bonferroni correction for multiple paired comparisons).

In addition, we measured the localization of the HA-tagged proteins inside the ‘recipient’ cells using the Max Contour Position score (location of the contour in the cell that has the highest intensity concentration, mapped to a number between 0 - the object center and 1 - the object perimeter)^40^. The distinct intracellular distribution of TDP-43 and α-synuclein after three days of co-culturing is demonstrated in Fig. 4B. There was a significant difference in the Max Contour Position between the two transmitted proteins. It is apparent that post transmission, α-synuclein is localized mainly in the periphery of the ‘recipient’ cells whereas TDP-43 is located closer to the center of the cell (P = 0.002, t-test). The unique distribution pattern of the two proteins in the ‘recipient’ cells is shown in Fig. 4C.

We also aimed to validate the assumption that the driving force for the transmission of α-synuclein and TDP-43 is a specific molecular behavior of the proteins and not of the tags or another unrelated effect. For that purpose, we co-cultured GFP-expressing cells with naïve SH-SY5Y cells for three days and monitored whether GFP is transferred to ‘recipient’ cells as compared to ‘time point zero’. We collected 100,000 focused images of single naïve ‘recipient’ cells and quantified the number of GFP^+^ cases. The naïve cells were gated for positive GFP signal using the Intensity and Max Pixel features. This fixed gate (termed: GFP^+^) defined the cell population in which GFP signal was detected (Supplementary Fig. S3). Next, in order to focus only on the internalized GFP, the cells were further gated according to two additional features: the Internalization and Max Contour Position scores (Supplementary Fig. S3). We found that there was no significant difference between GFP internalization at ‘time point zero’ and after three day co-culturing (0.71% ± 0.48 versus 0.55% ± 0.47 respectively, P = 0.59). We assume that this observation reflects the ‘background noise’ that the single cell imaging and the ability to set correct gating based on the imaging, is able to overcome. Representative single images of the internalized and non-internalized GFP in the naïve cells are presented in Supplementary Fig. S3.

**Figure 5:**
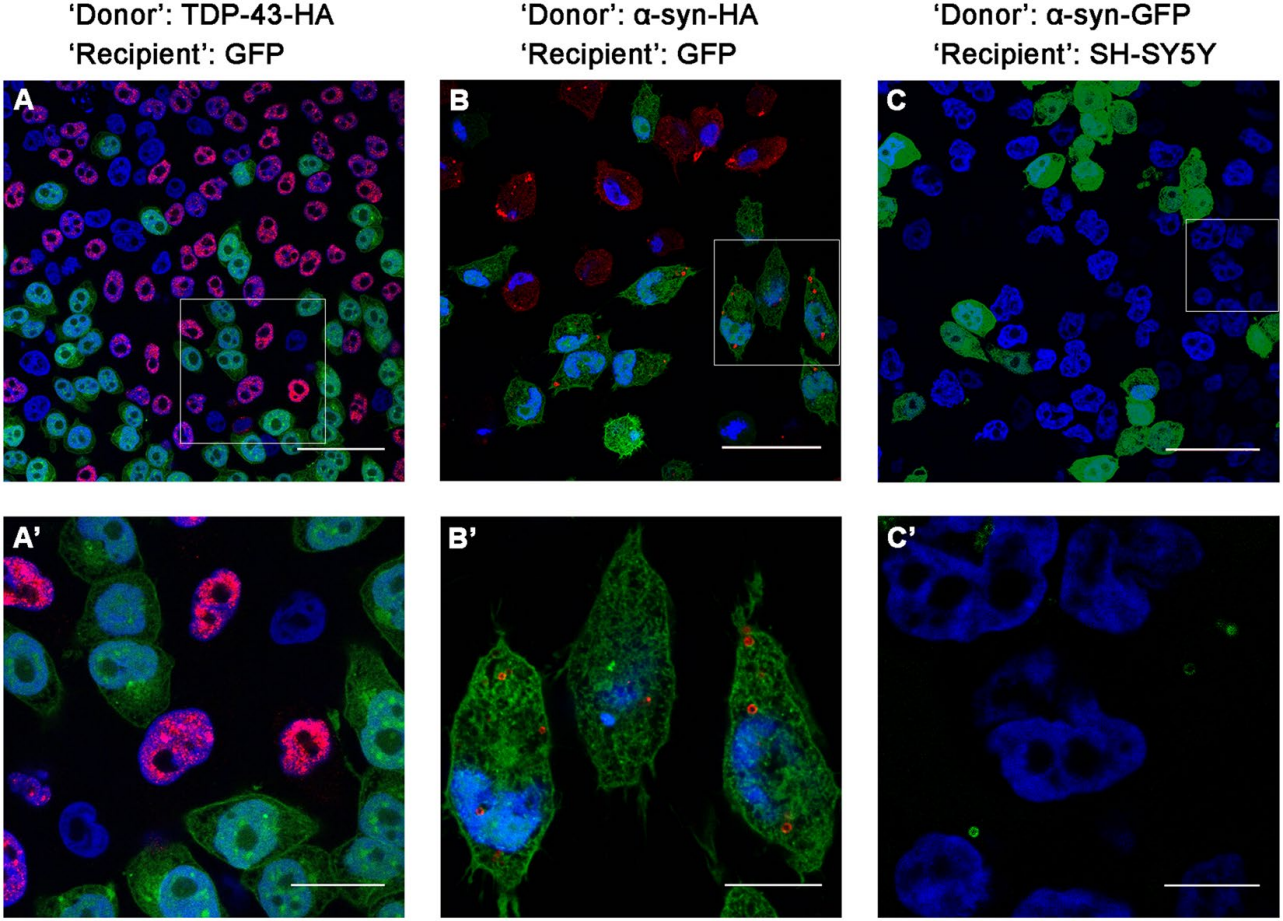
Transmission of tagged TDP-43 and α-synuclein into ‘recipient’ cells. The boxed area in (**A**,**B**,**C**) is enlarged in (**A’**,**B’**,**C’**), respectively. GFP-expressing ‘recipient’ cells were co-cultured with either HA-tagged TDP-43 (**A**,**A’**) or α-synuclein (**B**,**B’**) ‘donor’ cells for three days and viewed using confocal microscopy. Alternatively, ‘donor’ cells expressing GFP-tagged α-synuclein were co-cultured with naïve SH-SY5Y ‘recipient’ cells (**C**,**C’**). DAPI (blue), anti-HA-antibody (red). Scale bars: (**A**) (50 µm); (**A’**) (20 µm); (**B**,**C**) (40 µm); (**B’**) (12 µm); (**C’**) (8 µm).

As a complementary approach, we also monitored cell-to-cell transmission of the proteins using confocal microscopy. Similar conditions as for the IFC assay were employed and the cells were monitored for transfer events. Using this method, we were unable to detect TDP-43 transmission to the ‘recipient’ cells (Fig. 5A,A’). However, we specifically detected stained puncta of α-synuclein in many of the ‘recipient’ GFP cells (Fig. 5B,B’). Possible explanation for the difference between the two proteins will be provided in the Discussion.

We also co-cultured GFP-tagged α-synuclein with naïve SH-SY5Y cells and examined them using confocal microscopy. Similarly to HA-tagged α-synuclein, we observed the transmission of the GFP-tagged α-synuclein in vesicle-like puncta, though to a much lesser extent than HA-tagged α-synuclein, despite the fact that the cells expressing GFP-tagged α-synuclein were co-cultured for a longer period of time (six days compared to three days) (Fig. 5C,C’). This observation underscores the use of the HA-labeling system for monitoring cell-to-cell transmission.

**Figure 6:**
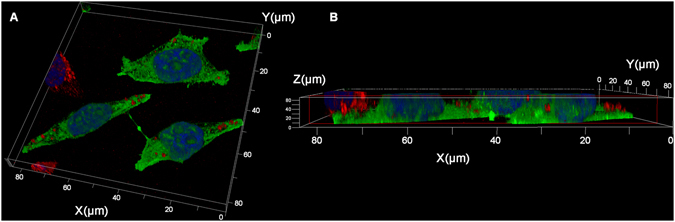
Three-dimensional localization of HA-tagged α-synuclein in GFP-expressing ‘recipient’ cells. GFP-expressing cells were co-cultured with ‘donor’ cells expressing HA-tagged α-synuclein for three days and 3D volume reconstruction of the Z-series (**A**) with XZ-slice projection (**B**) was performed. DAPI (blue), anti-HA-antibody (red). The interval between individual Z-stack serial images is 0.3 µm.

To investigate the exact localization of HA-tagged α-synuclein aggregates in the ‘recipient’ GFP cells, we used Z-stack and 3D reconstruction analysis. We found that the stained puncta were not restricted to the intracellular compartment. Importantly, in the majority of the cases, transferred α-synuclein was found outside of the cell milieu, attached to the outer membrane (Fig. 6). The results of the confocal images from the co-culturing experiments combined with their Z-stack analysis highlight the power of IFC for quantifying genuine cell-to-cell propagation in the current cell culture model. With the high throughput capacity of the single cell IFC we were able to include in our analysis only the cells in which HA-tagged puncta were exclusively intracellular.

Ultimately, we were also interested in identifying the aggregation state of the investigated proteins, TDP-43 and α-synuclein. For that purpose, we stained the cells using ThS, a dye that is well established for the detection of amyloid fibrils^41^. As a complementary approach, we also used immuno-staining with OC^42^, a specific antibody that detects amyloid fibrils as well, but not any pre-fibrillar or oligomeric structures. As a positive control, we supplemented the cells with recombinant α-synuclein pre-formed fibrils fluorescently labeled with Alexa488. We found that while ThS and OC did not stain the HA-tagged proteins, they did stain the Alexa488-labeled fibrils, as demonstrated in Supplementary Fig. S4. Therefore, we strongly believe that both over-expressed proteins are not found in a fibrillar structure in the cells.

Finally, we examined the aggregation state of TDP-43 and α-synuclein in samples of cell lysates. We isolated the insoluble fraction (protocol was modified from Bozzo *et al*.^43^) and found that in cells expressing HA-tagged TDP-43, TDP-43 is monomeric and also found as high-molecular weight aggregates (Supplementary Fig. S1). HA-tagged α-synuclein high-molecular weight aggregates were not detected in the insoluble fraction, however a smear representing a wide range of oligomeric species could be observed in samples of cell lysates papered in native conditions (Supplementary Fig. S1).

## Discussion

It is now appreciated that examining cell-to-cell transmission of proteins associated with various neurodegenerative disorders is a key step towards the understanding of the progression of these complex diseases and may offer new avenues for therapy. Studying the process of intercellular propagation has been hampered by the lack of methods that provide robust quantitative data. We sought to monitor the process at a single cell resolution using a very small protein tag to avoid the likelihood of artifacts. We focused on two proteins associated with neurodegenerative pathologies, TDP-43 and α-synuclein. While it has been confirmed that α-synuclein is indeed amyloidogenic and spreads between cells in a prion-like fashion, it is still debatable whether TDP-43 shares similar characteristics^10, 11^.

Following over-expression of each protein as a HA fusion in neurons, we opted for multispectral IFC, which has a distinctive advantage, as it can capture individual images of each single cell and analyze cells based on the intensity, pattern and localization of their fluorescent signal. Unlike conventional microscopy, IFC can be utilized for rare biological events that are difficult to identify^44, 45^. We confirmed HA-tagged α-synuclein and TDP-43 transfer from ‘donor’ to naïve ‘recipient’ cells in a cell culture model. To the best of our knowledge, this is the first report regarding the quantification of cell-to-cell transfer of TDP-43 in a well-controlled cell culture system. Using image analysis, that facilitates the discarding of ‘false positive’ events, we demonstrated that the transmission level of TDP-43 is very low. In the case of α-synuclein, the actual transmission level appears to be lower than previously reported^19^. These findings were validated based on the quantification of GFP transmission to naïve cells and the observation that GFP does not propagate between the cells over time. Moreover, the parallel study of the two proteins and the difference in their molecular behavior provides an inherent internal control for our observations.

For α-synuclein, β-amyloid and tau, a substantial body of evidence supports the hypothesis of prion-like spreading^10, 11^, whereas only a handful of studies focus on TDP-43 spreading. Feiler *et al*.^33^ recently reported the exchange of TDP-43 between cell somata inside exosomes or microvesicles and demonstrated that microvesicular TDP-43 exerts higher toxicity than free TDP-43 in the recipient cells. As for α-synuclein, recent studies have described potential mechanisms for its cell-to-cell transmission. Secretion via exosome release^46^, clathrin-mediated endocytosis^47^, lymphocyte-activation gene 3-mediated endocytosis^48^ and lysosomal vesicles traveling through tunneling nanotubes^49^ have been proposed to contribute to α-synuclein propagation. Specifically, it was demonstrated that α-synuclein aggregates move through the endosomal pathway and accumulate in lysosomes^50^. In addition, it was shown that α-synuclein induces the clustering of synaptic vesicles^51^. To conclude, our experimental data is consistent with the current information regarding TDP-43 and α-synuclein propagation properties^52, 53^, while providing an accurate and high resolution quantification of the process. The fact that we could detect differences and specificity in the transfer occurrence, as well as characteristic distribution patterns of the two distinct proteins, validates this original method which can be used for clarifying important biological questions.

As an additional approach for visualizing cell-to-cell transfer, we used confocal microscopy. The confocal screen of hundreds of cells for HA-tagged TDP-43 transfer indicated no detectable events. This is consistent with the low occurrence of cell-to-cell propagation of this protein as visualized by the IFC analysis of 100,000 single cells. Interestingly, confocal microscopy results of HA-tagged α-synuclein revealed some apparent transfer events to ‘recipient’ cells. However, 3D analysis indicated that most of the observed HA-tagged α-synuclein staining was at the cell periphery, which may not represent genuine cell-to-cell transfer. The observed difference between the two proteins may be the result of their differential clustering and secretion. We suggest that the observed transmission events, detected by confocal microscopy in the case of α-synuclein, which are more frequent than expected according to the IFC analysis, reflect the extracellular attachment of secreted vesicular assemblies of the protein.

We also characterized the aggregation state of both proteins and found that both HA-tagged TDP-43 and α-synuclein do not form fibrillar assemblies in our cell culture system. Moreover, we were able to detect oligomeric aggregates, along with the monomeric protein in neurons over-expressing HA-tagged TDP-43 and α-synuclein. Regarding this result, it has been confirmed by Domert *et al*.^54^ that either monomeric, oligomeric or fibrillar species of α-synuclein can be transferred to acceptor-cell population in co-culture^54^.

Taken together, our results demonstrate that investigating the intercellular propagation of α-synuclein and TDP-43 at the single cell level using multispectral IFC has several advantages over existing approaches. This newly established method will allow the examination of protein transfer while using inhibitors of exocytosis or aggregation. This will not only provide important insights regarding the spreading mechanism of pathological proteins, but will also facilitate the identification of specific targets for future drug development.

## Methods

### Cell lines

To generate SH-SY5Y (ATCC CRL-2266) cells stably expressing HA-tagged proteins, we used a CMV lentiviral system. We transfected HEK 293T cells (ATCC CRL-3216) with a transfection mixture containing 454 µL HeBS buffer (137.5 mM NaCl, l5 mM KCl, 7.5 mM Dextrose and 21 mM Hepes, pH 7.0), 9 µL PO_4_ (14 mM Na_2_HPO_4_), 41 μg total plasmid DNA (18.1 μg pCMV-α-synuclein-HA/TDP-43-HA or pCMV-GFP, 4.5 μg REV, 6 μg VSV.G and 11.8 μg MDL), H_2_O (to 900 µL) and 57 µL CaCl_2_ (2 mM). 48 hours post transfection, HEK 293T virus-containing medium was collected and used to infect naïve SH-SY5Y cells. The virus-containing medium was filtered via a 0.45 μm filter membrane and supplemented with 8 μg/mL polybrene. 5 μg/mL of puromycin were added 48 hours post infection to select for stably α-synuclein-HA/TDP-43-HA expressing SH-SY5Y cells. The infected cells were cultured in DMEM-F12 medium (Biological Industries) with supplements according to ATCC guidelines.

### Co-culture conditions and staining for flow cytometry

#### HA-tagged TDP-43/α-synuclein - Transmission experiments

We co-cultured neurons expressing HA-tagged α-synuclein/TDP-43 (‘donor’) with neurons expressing GFP (‘recipient’) in 1:1 ratio in 10 cm plates (10^6^ cells/plate) in DMEM-F12 growth medium for one day or three days at 37 °C with 5% CO_2_. The mixed cultures were harvested using trypsinization, and washed twice in Phosphate Buffered Saline (PBS). Cells were centrifuged and fixed in 4% paraformaldehyde (PFA) for 10 minutes at 37 °C. The cells were washed with PBS and treated with 90% methanol for 30 minutes on ice to allow cellular permeabilization. After washing the cells thoroughly, cells were blocked by washing twice with 0.5% BSA followed by 30 minute incubation. Then, the cells were stained using a mouse monoclonal anti-HA antibody (sc-7392, Santa Cruz) in 1:200 ratio in blocking solution, for 1 hour at room temperature. The cells were washed again three times with PBS and then stained with a secondary, anti-mouse Cy5 antibody (#115175146, Jackson Immunoresearch) in 1:200 ratio in blocking solution for 30 minutes at room temperature. Cells were washed three times, and pellets were resuspended in PBS to a final concentration of ~2 × 10^6^ cells/50 µL. DAPI staining (Sigma-Aldrich) was added prior to cell analysis. In order to prepare ‘time point zero’ samples, ‘donor’ cells and ‘recipient’ cells were cultured separately, fixed, and then mixed in 1:1 ratio before permeabilization and staining.

#### GFP to naïve cells – Control experiment

We co-cultured neurons expressing GFP (‘donor’) with naïve SH-SY5Y neurons (‘recipient’) in 1:1 ratio in 10 cm plates (10^6^ cells/plate) in DMEM-F12 growth medium for three days at 37 °C with 5% CO_2_. The mixed cultures were harvested using trypsinization, and washed twice in PBS. Cells were centrifuged and fixed in 4% PFA for 10 minutes at 37 °C. The cells were washed with PBS and treated with 90% methanol for 30 minutes on ice to allow cellular permeabilization. Cells were washed three times with PBS, and pellets were resuspended in PBS to a final concentration of ~2 × 10^6^ cells/50 µL. DAPI staining was added prior to cell analysis. In order to prepare ‘time point zero’ samples, ‘donor’ cells and ‘recipient’ cells were cultured separately, fixed, and then mixed in 1:1 ratio before fixation and permeabilization.

### Multispectral imaging flow cytometry (ImageStreamX) analysis

Cell were co-cultured as described above and imaged using multispectral imaging flow cytometry (ImageStreamX markII flow cytometer; Amnis Corp, part of EMD millipore, Seattle, WA). Laser settings were 405 nm = 30 mW, 488 nm = 2.5 mW, 642 nm = 45 mW. At least 10^5^ focused images of single ‘recipient’ cells were collected from each sample. The pattern and intensity of the ‘recipient’ cells served as a reference for defining the physical parameters for gating the correct cell population. In the case of the transmission experiments, it allowed us to validate that only true GFP positive cells were included (and not GFP transferred to ‘donors’ cells).

Data were analyzed using the manufacturer’s image analysis software (IDEAS 6.2; Amnis Corp). Images were compensated for fluorescent overlap by using each cell line as a control. Cells were gated for single cells using the area and aspect ratio features, and for focused cells using the Gradient RMS feature^55^. To eliminate dead cells and debris, cells were first gated for positive nuclear staining, using the area and aspect ratio features of the DAPI labeled nuclear image.

#### HA-tagged α-synuclein/TDP-43 - Transmission experiments

Evaluation of protein transfer was done by gating GFP positive cells for Cy5 staining using two features, the Intensity (the sum of the background subtracted pixel values within the image) and the Max Pixel (the largest value of the background-subtracted pixels contained in the image). To further eliminate events in which the Cy5 staining originated from cell debris outside the cell, we used two additional features: the Similarity score between the GFP and the Cy5 staining (the log transformed Pearson’s correlation coefficient in the two input images), and the Internalization score (the ratio of the intensity inside the cell to the intensity of the entire cell). The inner cell mask for this purpose was defined using the Morphology mask (includes all pixels within the outermost image contour, used for calculating the values of overall shape-based features) of the GFP channel. Cells with low similarity and low internalization values were excluded from the analysis. The differential localization of HA-tagged TDP-43 and α-synuclein inside the cell was quantified using the Max Contour Position score (the location of the contour in the cell that has the highest intensity concentration, mapped to a number between 0 - the object center and 1 - the object perimeter).

#### GFP to naïve cells – Control experiment

Evaluation of protein transfer was done by gating naïve SH-SY5Y cells for GFP staining using two features, the Intensity and the Max Pixel. In order to focus only on the internalized GFP, the cells were gated according to two additional features: the Internalization and Max Contour Position scores.

### Statistical analysis

Data were analyzed with the Minitab Software, version 16 (Minitab Inc, State College, PA). Staining over time was analyzed using repeated-measures analysis of variance followed by Bonferroni correction for multiple paired comparisons (two-sided). Student t-test was used to compare Max Contour Position between groups on day three. A p-value of less than 0.05 was considered statistically significant.

### Co-culture conditions and immuno-staining for confocal microscopy

We co-cultured neurons expressing HA-tagged TDP-43/α-synuclein (‘donor’) with neurons expressing GFP (‘recipient’) at 1:1 ratio in 6-well plate (0.3 × 10^6^ cells/well) on glass slides coated with 0.01% Poly-L-Lysine (Sigma-Aldrich). Cells were co-incubated for three days and then washed and fixed in 4% PFA for 15 minutes at room temperature. The cells were washed twice with PBS and treated with 0.25% Triton (Sigma-Aldrich) for 10 minutes at room temperature to allow cellular permeabilization. After washing the cells thoroughly, they were blocked with 1% BSA for 30 minutes at room temperature. Then, we directly stained the cells using a mouse monoclonal anti-HA antibody (sc-7392, Santa Cruz) in a 1:200 ratio in blocking solution, for 1 hour at room temperature. Slides were washed again three times with PBS and a secondary, anti-mouse Cy3 antibody (ab97035, abcam) was added in a 1:200 ratio in blocking solution for another 30 minutes at room temperature. Finally, cells were washed three times and the glass slides were moved to cover slide with 15 µL VECTASHIELD antifade mounting medium containing DAPI (H-1500, Vector laboratories) to allow staining of the nuclei. Imaging was performed using SP8 inverted confocal microscopy (Leica Microsystems, Wetzlar, Germany) equipped with a Leica HC PL APO CS2 × 63/1.4 NA objective. Excitation and emission ranges: DAPI (412–450), GFP (495–519), Cy3 (548–561). 3D reconstructions were performed in LAS-AF software.

### Total cell lysates preparation and western blotting

8 × 10^6^ cells were harvested using trypsinization, collected by centrifugation and washed in PBS. Each sample was lysed in 500 μL RIPA buffer (50 mM Tris-HCl, pH 8.0, 150 mM NaCl, 1% NP-40, 0.5% sodium deoxycholate, 0.1% SDS) containing a protease inhibitor cocktail (Roche) and 4 M Urea. After 30 minutes of incubation on ice, lysates were centrifuged at 13,500 rpm for 30 minutes at 4 °C. The supernatants were collected and protein concentration was determined using Bradford protein assay (Bio-rad). Lysates were heated to 100 °C for 10 minutes in sample buffer containing β-mercaptoethanol. Samples were resolved on SDS–PAGE and transferred to PVDF membrane using iBlot gel transfer stacks (Invitrogen). Membranes were fixed in 4% PFA, blocked (1 hour in 5% nonfat milk in Tris Buffered Saline (TBS), 0.1% Tween-20 (TBS-T)) and incubated overnight at 4 °C with the indicated antibodies diluted in 5% nonfat milk in TBS-T. Immuno-reactive TDP-43 was detected with a rabbit polyclonal anti-TDP-43 antibody in 1:1000 ratio (10782–2-AP, Proteintec). Immuno-reactive α-synuclein was detected with a rabbit polyclonal anti-α-synuclein antibody in 1:1000 ratio (sc-7011, Santa cruz). For detecting actin, mouse monoclonal anti-β-actin in 1:5000 ratio (ab8224, abcam) was used; after washing twice with TBS-T and once with TBS, membranes were incubated with the appropriate peroxidase-conjugated secondary antibody in 5% nonfat milk in TBS-T for 45 minutes. The membrane was developed using Western HRP substrate (Millipore) according to manufacturer’s instructions, developed and exposed to Fuji Medical X-Ray Film for up to over-night. Films were developed using Kodak X-OMAT 2000.

### TDP-43 and α-synuclein aggregation assay

4 × 10^5^ cells were scraped off the plate in culture medium, collected by centrifugation, washed in PBS and split to two samples. Each sample was lysed in 100 μL high salt RIPA buffer (50 mM Tris-HCl pH 8.0, 5 mM EDTA pH 8.0, 250 mM NaCl, 1% Triton X-100, 0.25% sodium deoxycholate, 0.1% SDS) containing a protease inhibitor cocktail (Roche). After 20 minutes of incubation on ice, lysates were centrifuged at 20,000 × g for 10 minutes at 4 °C. The supernatants were collected as the soluble fraction, whereas the pellets (insoluble fractions) were washed in RIPA buffer and resuspended in 100 μL of Laemmli sample buffer (62 mM Tris-HCl pH 6.8, 10% glycerol, 2% SDS, 0.05% bromphenol blue) without β-mercaptoethanol. Protein concentration of soluble fractions was determined using Bradford protein assay (Bio-rad) and values obtained were used to indirectly quantify the insoluble fractions (the protocol was modified from Bozzo *et al*.^43^). Samples were further analyzed by western blotting in similar conditions and using the same antibodies as described above (‘Total cell lysates preparation and western blotting’ section).

### Native PAGE

8 × 10^5^ cells were scraped off the plate in culture medium, collected by centrifugation, washed in PBS. Lysates were obtained using sonication in detergent-free lysis buffer (50 mM Tris/HCl pH 7.4, 175 Mm NaCl, 5 mM EDTA pH 8.0 and protease inhibitor cocktail (Roche)). Lysates were centrifuged at 13,500 rpm for 30 minutes at 4 °C, the supernatants were collected and protein concentration was determined using Bradford protein assay (Bio-rad). 20 µg of each lysate in native sample buffer (Serva) was loaded on the gel and run with detergent-free running buffer (GeneScript). Samples were further analyzed by western blotting in similar conditions and using the same antibodies as described above (‘Total cell lysates preparation and western blotting’ section). The protocol was modified from Outeiro *et al*.^56^.

### ThS Staining

Cells were grown in 24-well plate on glass slides coated with 0.01% Poly-L-Lysine. When reaching ~30–40% confluency, cells were treated with 500 µL of Opti-MEM containing 10 µg of pre-formed Alexa488-labled α-synuclein fibrils and 6 µL of Lipofectamine LTX transfection reagent (ThermoFisher Scientific) for 3 hours at 37 °C. The medium was replaced to DMEM-F12 and cultured for additional 14 hours. Cells were washed once with trypsin/EDTA to remove extracellular α-synuclein fibrils and then washed with PBS, fixed for 30 minutes in 4% PFA and treated with 0.25% Triton for 10 minutes at room temperature to allow cellular permeabilization. After two washes with PBS, the cells were incubated with ThS (0.025%) for 8 minutes. Later the samples were washed three times in 80% ethanol and twice in H_2_O. After washing the cells thoroughly with PBS, they were blocked with 1% BSA for 30 minutes at room temperature. Then, we directly stained the cells using a mouse monoclonal anti-HA antibody (sc-7392, Santa Cruz) in a 1:200 ratio in blocking solution, for 1 hour at room temperature. Slides were washed again three times with PBS and a secondary anti-mouse Cy5 antibody (#115175146, Jackson Immunoresearch) was added in a 1:200 ratio in blocking solution for 30 minutes at room temperature. To complete the sample preparation, we followed the protocol described above (‘Immuno-staining for confocal microscopy’ section). Excitation and emission ranges: DAPI (412–450), 488 (495–519), Ths (524–575), Cy5 (647–665).

### OC antibody immuno-staining

Cells were grown in 24-well plate on glass slides coated with 0.01% Poly-L-Lysine. When reaching ~30–40% confluency, cells were treated with 500 µL of Opti-MEM containing 10 µg of pre-formed Alexa488-labled α-synuclein fibrils and 6 µL of Lipofectamine LTX for 3 hours at 37 °C. The medium was replaced to DMEM-F12 and culture was continued for 14 hours. Cells were washed once with trypsin/EDTA to remove extracellular α-synuclein fibrils. Cells were washed with PBS, fixed and permeabilized as previously described (‘immuno-staining for confocal microscopy’ section). Later, cells were blocked with 1% BSA for 30 minutes and stained with the rabbit-polyclonal OC antibody (ab2286, millipore) and with a mouse-monoclonal anti-HA antibody (sc-7392, Santa Cruz) in a 1:200 ratio in blocking solution, for 1 hour at room temperature. Slides were washed again three times with PBS and then two secondary antibodies were added: anti-mouse Cy5 antibody (#115175146, Jackson Immunoresearch) and anti-rabbit Cy3 antibody (#111165003, Jackson Immunoresearch) were added in a 1:200 ratio for another 30 minutes at room temperature. To complete the sample preparation, we followed the protocol described above (‘Immuno-staining for confocal microscopy’ section). Excitation and emission ranges: DAPI (412–450), 488 (495–519), Cy3 (548–561), Cy5 (647–665).

## Electronic supplementary material

Supplementary information
