# Supplementary material for: Single cell imaging and quantification of TDP-43 and α-synuclein intercellular propagation
Source: Sci Rep. 2017 Mar 28;7:544. doi: 10.1038/s41598-017-00657-z (PMC5428807; doi:10.1038/s41598-017-00657-z)
Supplement: Supplementary file 1 — Supplementary information [file 41598_2017_657_MOESM1_ESM.pdf]

Single cell imaging and quantification of TDP-43 and α-synuclein intercellular propagation

Sivan Peled[1,§], Dorin Sade[1,§], Yaron Bram[1,¥], Ziv Porat[2], Topaz Kreiser[1], Michael Mimouni[3], Alexandra Lichtenstein[4], Daniel Segal[1,5], Ehud Gazit[1,5,*]

[1]Department of Molecular Microbiology and Biotechnology, Tel-Aviv University, Tel Aviv, 6997801, Israel.

[2]Flow Cytometry Unit, Department of Biological Services, Weizmann Institute of Science, Rehovot, Israel.

[3]Department of Ophthalmology, Rambam Health Care Campus, Haifa, Israel.

[4]Sackler Cellular and Molecular Imaging Center, Sackler Faculty of Medicine, Tel-Aviv University, Israel.

[5]Sagol Interdisciplinary School of Neurosciences, Tel Aviv University, Tel Aviv, 69978, Israel.

[§]Both authors contributed equally to this work

*To whom correspondence should be addressed: ehudg@post.tau.ac.il

[¥]Current address: Division of Gastroenterology & Hepatology, Department of Medicine, Weill Medical College of Cornell University, New York, NY 10021, USA

Supplementary information

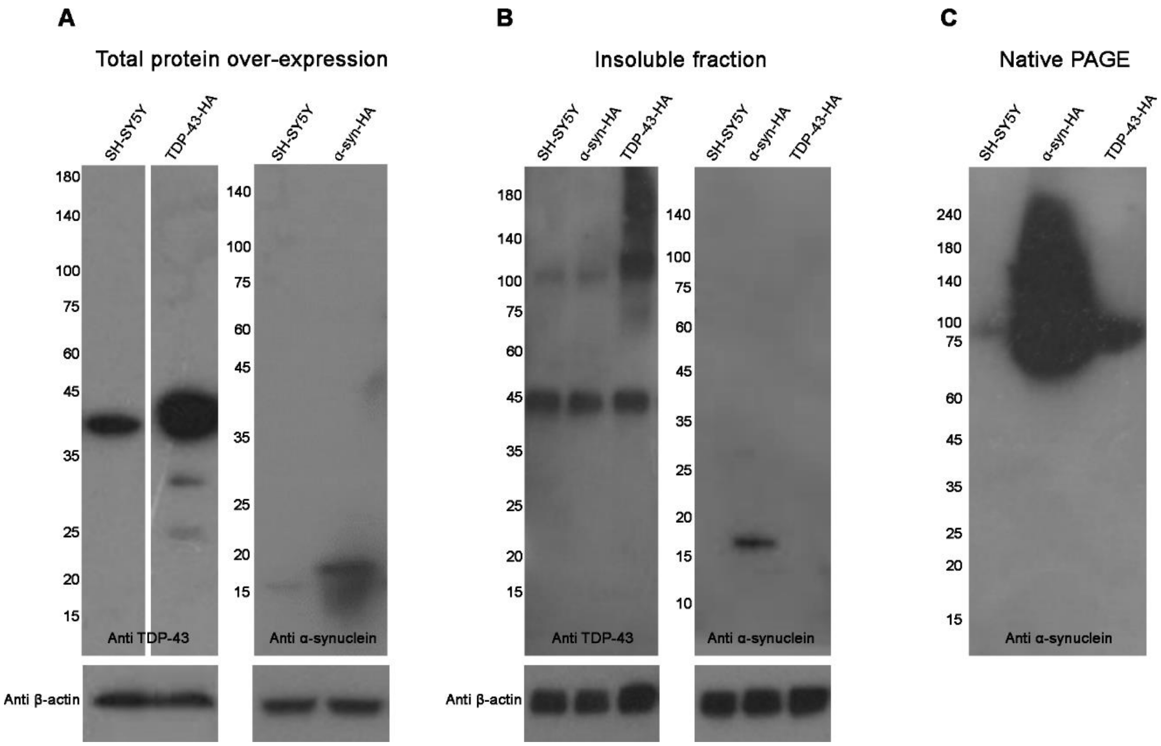

<u>Supplementary Figure S1</u>: Over-expression of tagged TDP-43 and α-synuclein. **A.** Cells were lysed in RIPA buffer with 4M Urea, subjected to SDS-PAGE, analyzed by western blot and immuno-reacted with anti-TDP-43 (left panel; lanes were vertically juxtapose from the same blot) or anti-α-synuclein antibody (right panel) and anti-β-actin antibody. Level of over-expressed proteins was quantified and estimated to be ~3 fold higher compared to endogenous protein level. Quantification was performed using ImageQuant™ TL. **B.** Cells were lysed in high salt RIPA buffer, pellets were collected and dissolved in Laemmli sample buffer and subjected to SDS-PAGE and analyzed by western blot and immuno-reacted with anti-TDP-43 (left panel) or anti-α-synuclein antibody (right panel) and anti-β-actin antibody. We were able to detect high-molecular weight aggregates only in the HA-tagged TDP-43 cells and not in the HA-tagged α-synuclein cells. **C.** Cells were sonicated in detergent-free buffer, subjected to native PAGE and immuno-reacted with anti-α-synuclein antibody. The smear in the lysates obtained from cells over-expressing HA-tagged α-synuclein are indicative of a wide range of oligomeric species (see Materials and Methods).

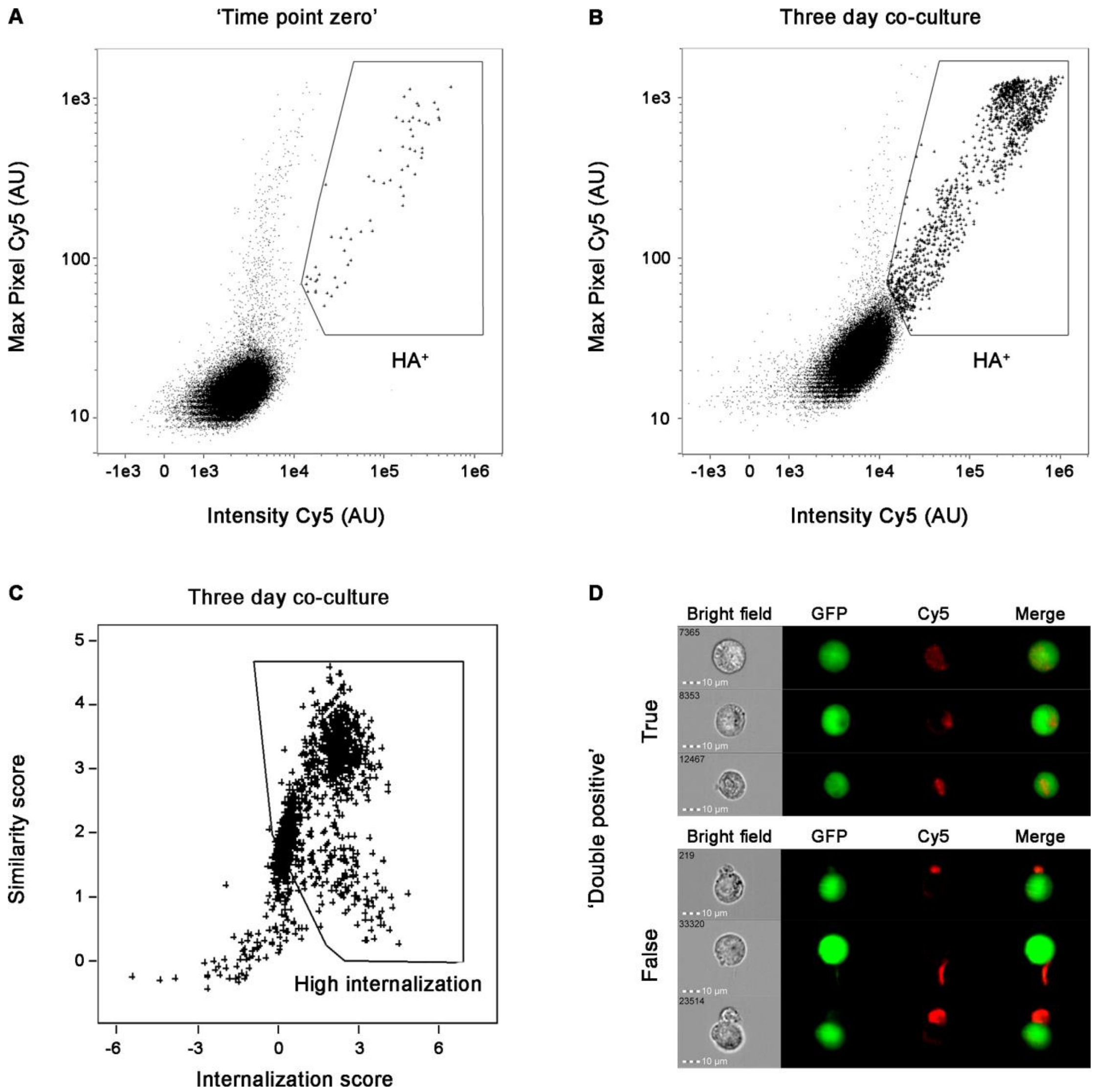

<u>Supplementary Figure S2</u>: IFC analysis of GFP-expressing 'recipient' cells co-cultured with 'donor' cells expressing HA-tagged TDP-43. **A-B.** Bivariate analysis of GFP-expressing 'recipient' cells collected from (**A**) 'time point zero' and (**B**) three day co-culture. HA^+ gated cells are demarcated. **C.** Double positive (HA^+) cells collected after three days co-culture were further gated to exclude Cy5 staining outside cell boundaries using the Internalization score and the Similarity score between the GFP and Cy5 channels (see Materials and Methods). The gated cells represent a high Internalization score within the GFP-expressing cells .**D.** Images representing true and false 'double positive' cells.

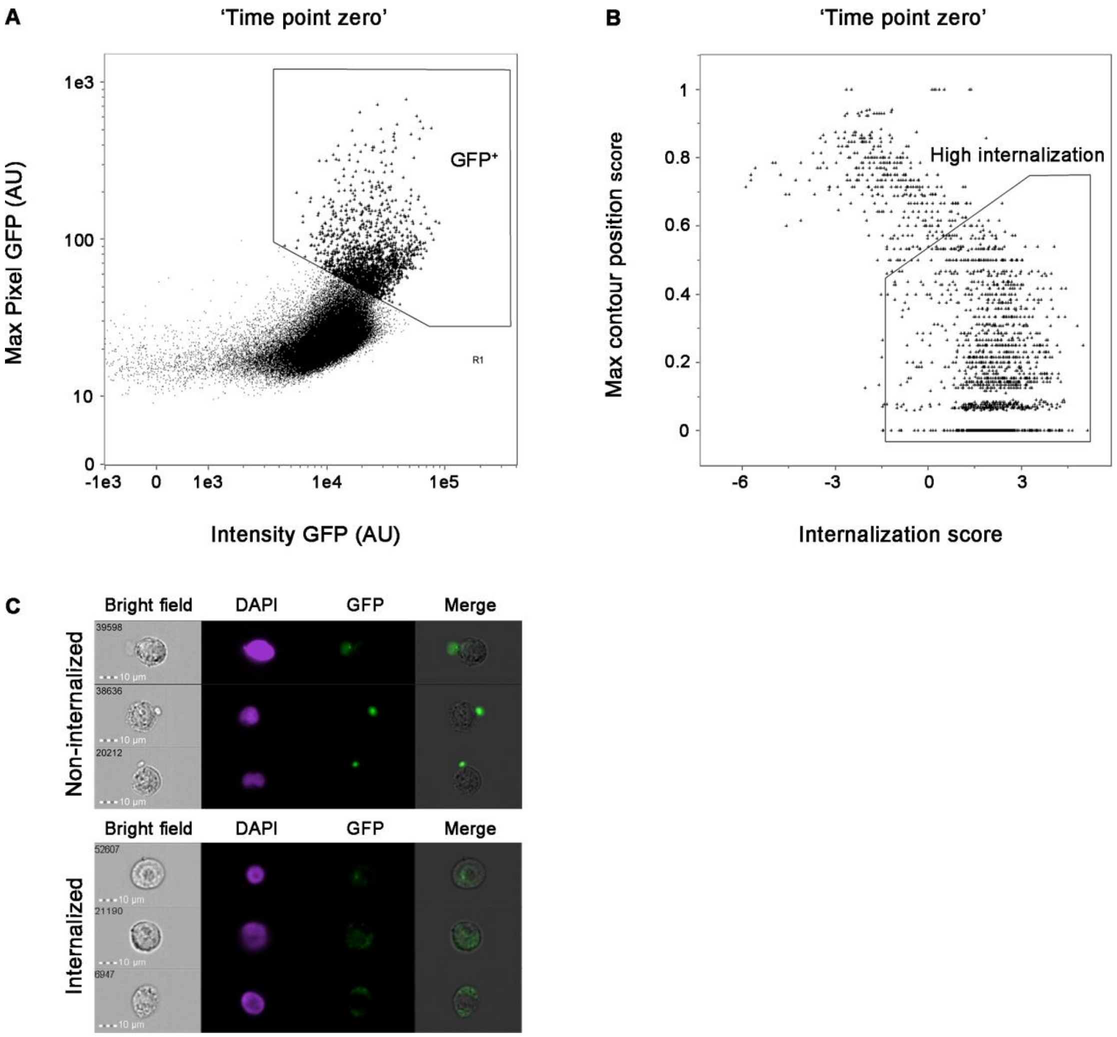

Supplementary Figure S3: IFC analysis of naïve 'recipient' cells co-cultured with GFP-expressing 'donor' cells. **A.** Bivariate analysis of naïve 'recipient' cells collected from 'time point zero'. GFP^+ gated cells are demarcated. **B.** Positive (GFP^+) cells collected were further gated to exclude GFP signal residing outside cell boundaries using the Internalization score and the Max contour position score (see Materials and Methods). The gated cells represent a high Internalization score within the naïve cell population. **C.** Images representing 'Internalized' and 'Non-internalized' GFP transfer to naïve cells.

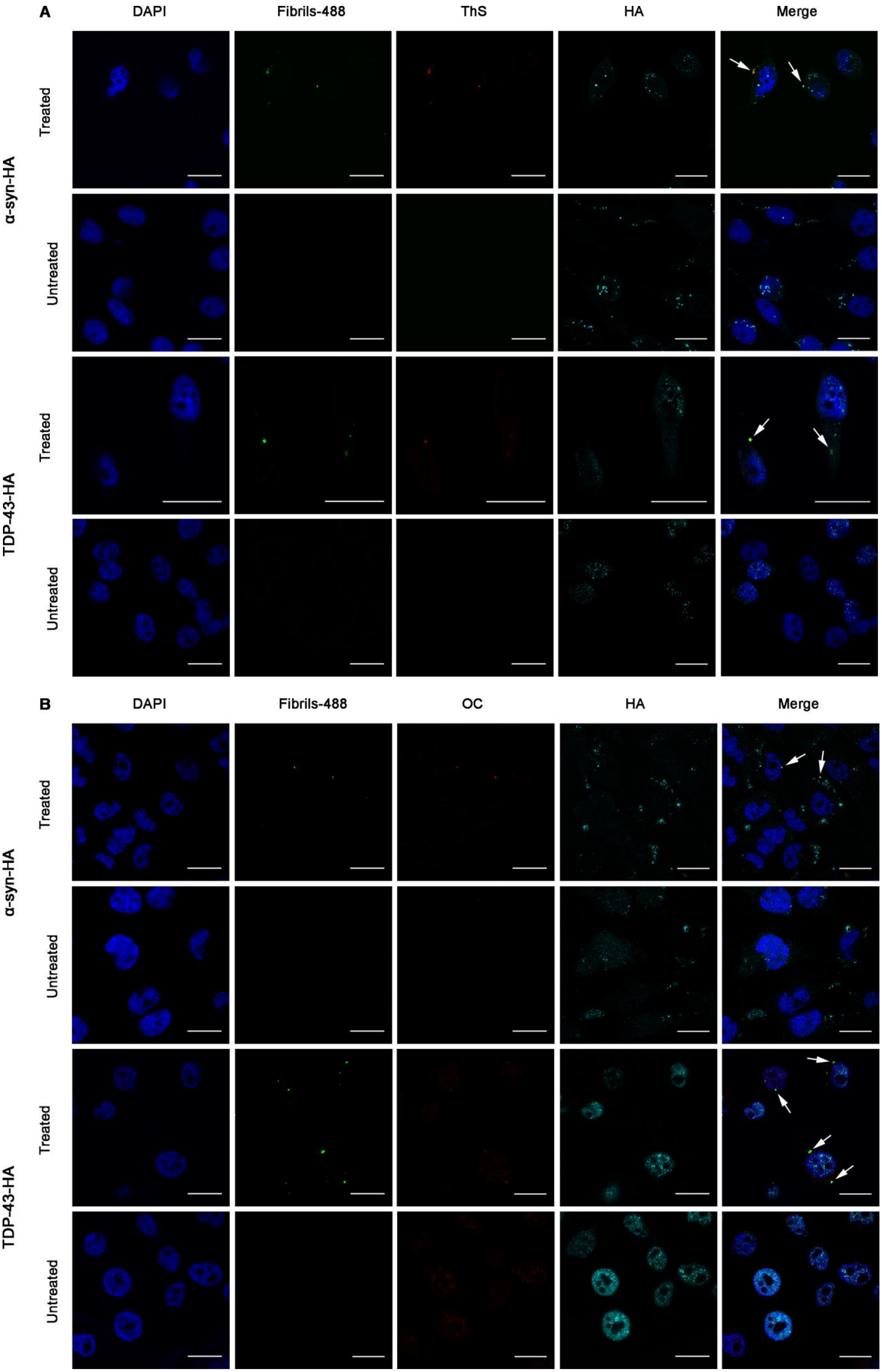
A
DAPI
Fibrils-488
ThS
HA
Merge
α-syn-HA
Treated
Untreated
TDP-43-HA
Treated
Untreated
B
DAPI
Fibrils-488
OC
HA
Merge
α-syn-HA
Treated
Untreated
TDP-43-HA
Treated
Untreated

Supplementary Figure S4: Scheme of the studied proteins (α-synuclein and TDP-43) stained with ThS (**A.**) and OC antibody (**B.**). ThS and OC antibody stain recombinant fibrils-488 (see arrows) however they do not stain or co-localize with the HA-tagged proteins. DAPI (blue), Fibrils-488 (Green), ThS/OC (red) and anti-HA-antibody (turquoise). Scale bar: 20 μm.
